# Public awareness of income-related health inequalities in Ontario, Canada

**DOI:** 10.1186/1475-9276-11-26

**Published:** 2012-05-21

**Authors:** Ketan Shankardass, Aisha Lofters, Maritt Kirst, Carlos Quiñonez

**Affiliations:** 1 Centre for Research on Inner City Health, St. Michael’s Hospital, Toronto, ON, Canada; 2 Department of Psychology, Wilfrid Laurier University, 75 University Avenue, Waterloo, ON, N2L 3C5, Canada; 3 St. Michael’s Department of Family & Community Medicine, University of Toronto, Toronto, ON, Canada; 4 Ontario Tobacco Research Unit, Dalla Lana School of Public Health, University of Toronto, Toronto, ON, Canada; 5 Faculty of Dentistry, University of Toronto, Toronto, ON, Canada

**Keywords:** Health equity, Health inequalities, Public opinion, Public awareness, Media, Political will

## Abstract

**Introduction:**

Continued action is needed to tackle health inequalities in Canada, as those of lower income continue to be at higher risk for a range of negative health outcomes. There is arguably a lack of political will to implement policy change in this respect. As a result, we investigated public awareness of income-related health inequalities in a generally representative sample of Ontarians in late 2010.

**Methods:**

Data were collected from 2,006 Ontario adults using a telephone survey. The survey asked participants to agree or disagree with various statements asserting that there are or are not health inequalities in general and by income in Ontario, including questions pertaining to nine specific conditions for which inequalities have been described in Ontario. A multi-stage process using binary logistic regression determined whether awareness of health inequalities differed between participant subgroups.

**Results:**

Almost 73% of this sample of Ontarians agreed with the general premise that not all people are equally healthy in Ontario, but fewer participants were aware of health inequalities between the rich and the poor (53%–64%, depending on the framing of the question). Awareness of income-related inequalities in specific outcomes was considerably lower, ranging from 18% for accidents to 35% for obesity.

**Conclusions:**

This is the first province-wide study in Canada, and the first in Ontario, to explore public awareness on health inequalities. Given that political will is shaped by public awareness and opinion, these results suggest that greater awareness may be required to move the health equity agenda forward in Ontario. There is a need for health equity advocates, physicians and researchers to increase the effectiveness of knowledge translation activities for studies that identify and explore health inequalities.

## Introduction

Recent evidence indicates that continued and greater action is needed to tackle health inequalities in Ontario, Canada’s most populous province^a^. Ontarians of lower income are at higher risk for heart disease [1], poor mental health [2], stress and anxiety [3], depression [4] alcoholism [5], accidents [6], diabetes [7], obesity [8], lung cancer [9], and a range of other negative outcomes [10,11]. While the creation of 14 Local Health Integration Networks in 2005 by the Ontario Ministry of Health and Long-Term Care partly reflected an attempt by the Ontario government to create a more equitable health care system [12], there is little evidence of concerted effort at the provincial level to address factors outside of the health care sector that can prevent income-related health inequalities.

For example, affordable high-quality housing has long been identified as an important determinant of health and well-being, particularly for vulnerable populations (e.g., those of lower income, recent immigrants, the homeless and vulnerably housed) [13-17]. Yet, in spite of the long-standing recommendation from the Spasoff Report in 1987 that achieving health equity means that “society has a collective responsibility” (p. 45) to facilitate access to resources like affordable housing [18,19], there remains a significant under-supply of affordable housing in Ontario [13,20].^b^

We can consider the reasons for this and other shortcomings in pro-health equity policies in Ontario using Kingdon’s [21] notion that policy changes occur in “windows of opportunity” that are comprised of three analytically distinct “streams”: (1) the “problem stream”, i.e., are health inequalities identified as a concern in Ontario?, (2) the “policy stream”, i.e., are there solutions for health inequalities in Ontario?, and (3) the “political stream”, i.e., is there political will to foster greater health equity in Ontario? To the extent that income-related health inequalities (as described above) have been noticed by policy makers in Ontario, and that viable solutions are possible, the ongoing failure of the Canadian federal, provincial and municipal governments to act on the determinants of health outside of health care may partly reflect a lack of political will [22], which requires advocates and researchers to examine the drivers of political will on healthy equity.

In democracies like Canada, public opinion can be an important driver of political will on health and social issues. In particular, evidence from a retrospective analysis of policy making in Canada from 1994–2001 (i.e., the years that Jean Chrétien was Prime Minister) suggests that public opinion and public policy are more likely to be concordant in this country on higher profile issues (e.g., deficit reduction); that is, where the public is more aware of the issues requiring policy changes [23]. We view public awareness of health inequalities as one important determinant of public opinion on this topic. Several authors have discussed public opinion and awareness of social inequalities in health as a determinant of public support for government intervention [24-26]. In this way, we examined awareness of income-related health inequalities among Ontarians as a reflection of whether there can be enough political will to address these and other factors outside of the health care domain (e.g., housing) in ameliorating health inequalities. We concentrated on income-related inequalities for three reasons. First, pilot testing indicated that this was the most consistent way that participants conceived of and understood health differences between individuals, i.e. between “rich and poor” as opposed to between “educated and uneducated.” Second, it is arguable that income-related inequalities are the most amenable to policy intervention (see Bryant et al. 2011; [27]). Third, we tried to construct a survey that would be comparable to others in the literature, and others have surveyed public opinion about income-related health inequalities (e.g., [28]).

## Methods

Data were collected from a sample of 2,006 Ontarians aged 18 years and over in the fall and winter of 2010 through a telephone interview survey using random digit dialing (RDD). A sample size calculation indicated that this could provide a 3.0% margin of error with 95% confidence relative to the Ontario population. A market-based research firm (Opinion Search) was employed to administer the survey. Landline telephone numbers were randomly sampled, but quotas based on the Ontario population for sex, age, and geography (urban/rural) were imposed. Of 69,906 numbers called, there were 56,528 eligible calls (excluding numbers that were not in service, fax machines, or invalid). After exclusions for busy signals, answering machines, no answer, language barrier, ill or incapable participants, and eligible persons not available, a total of 33,530 individuals were asked to participate. Importantly, only one person was chosen from each household. Overall, a response rate of 5.49% was achieved, with 9.24% of persons asked to complete the survey doing so. Willingness to participate was taken to imply consent, and no personal identifiers were collected. Surveys were conducted in English. The study received approval by the University of Toronto’s Office of Research Ethics.

The survey included questions pertaining to three broad themes: (1) awareness of health inequalities; (2) attributions of the causes of health inequalities; and (3) opinions about possible solutions to health inequalities. Two groups of outcomes examined in this analysis relate to the first theme only. First, we analyzed responses to four questions that asked participants to agree or disagree with various statements asserting that there are or are not health inequalities in general and by income in Ontario, specifically: (*i*) In Ontario, all people are equally healthy and can expect to live for more or less the same amount of time; (*ii*) In Ontario, people who are rich are much healthier than those who are poor; (*iii*) In Ontario, people who are poor are less likely to live into their 80s than people who are rich; and (*iv*) Over the last few years, people who are rich have become healthier while people who are poor have become less healthy. For the purpose of analysis, we examined the proportion of participants agreeing that there are health inequalities in Ontario (i.e., question *i* was reverse coded). A second group of questions asked participants whether they felt that rich people were more, less or equally likely than poor people to suffer from nine conditions for which income-related inequalities have been described in Ontario, including: heart disease, mental illness, stress and anxiety, depression, accidents, diabetes, obesity, lung disease and alcoholism. In our analysis, we examined the proportion of participants that identified that the rich are less likely to suffer from these outcomes to facilitate comparison with the first set of questions. For all questions participants were given the option to respond with “don’t know” or to refuse to answer. The proportion answering “don’t know” or refusing to answer ranged from 1.2% to 7.4% with a mean of 2.9% and a standard deviation of 1.6%, and these responses were excluded from all analyses.

In order to investigate participant characteristics that may influence the likelihood of awareness of health inequalities, information was also collected on participant demographics, political affiliation, awareness of public health issues, and self-reported health status. Demographic characteristics included sex, age group (18–34 years, 35–54 years, 55+ years), urban or rural place of residence, immigration status (born in Canada, immigrated greater or equal to, or less than 10 years ago), language spoken most often at home (English or non-English) and ethnic ancestry to identify visible minority status (those who did not report Canadian, American or European, including Russian, ethnic ancestry). Low socioeconomic position was measured using three variables that were examined separately, including total household income in 2009 below $40,000, high school diploma or lower as the highest attained education, and unemployment at the time of the survey. Political affiliation was gauged in response to the question, “If the election were being held today, do you think you would vote for the Progressive Conservative, Liberal, New Democratic Party (NDP), Green, or some other candidate?” For the purpose of analysis, participants who indicated affiliation with the Green Party or “some other” candidate were classified as Other. The former three parties are the three parties currently represented in the Ontario legislative assembly. We also examined a fourth category of political affiliation that comprised participants who either didn’t know who they would vote for or who refused to answer the question. Public health awareness was gauged by asking participants to measure their “knowledge and understanding of the health issues affecting Ontarians” using a Likert scale (Poor, Fair, Good, Very good, Excellent); where we examined variation in the study population between those indicating a very good or excellent compared to worse understanding. Finally, we measured self-rated health using the same Likert scale, where we examined variation in the study population between those indicating fair or poor compared to better self-rated health.

We followed a multi-stage process to determine whether public awareness of health inequalities differed between participant subgroups. First, we systematically tabulated the proportion of participants that were aware of health inequalities for each question (i.e., agreed with statements implying inequalities or felt that the rich were less likely to suffer from a given outcome) across all participant subgroups and identified significant differences based on a chi-square test with an alpha level of 0.05. Second, we examined the Spearman correlation coefficient between all significant subgroup characteristics in order to identify potentially non-independent predictors of awareness for each question. Third, we built a binary logistic regression model for each question using a manual backward stepwise approach to identify a parsimonious list of subgroup characteristics that independently predicted relative *unawareness* of health inequalities.

For all analyses, data were weighted to replicate provincial population distributions, by age and sex, according to 2006 Census data. Statistical Package for the Social Sciences (SPSS) for Windows software (SPSS Inc., version 16.0) was used for all analyses.

## Results

Study participants were generally representative of the Ontario population based on the 2006 Census [29], although were better educated on average (only 26.5% with high school diploma or less as their highest attained education compared to 38%)^c^, and slightly less likely to live in a Census Metropolitan Area: a geographic area with an urban core whose population is at least 100,000 based on the 2006 Census (73% compared to 79%). When compared with Ontarians responding to the 2007 Canadian Community Health Survey [30], our participants were slightly more likely to report poor or fair self-rated health (14% compared to 11%).

A large majority of our participants (72.7%) lived in a CMA (Table 1). Females comprised 52% of the study population, 24% were foreign-born, and 17% were ethnic/cultural minorities. Almost 30% of participants reported an annual household income less than or equal to $40,000, while 26.5% reported a high school diploma (or less) as the highest attained degree, and 6.2% reported current unemployment. When asked who they would vote for if an election were being held today, 24.5% reported Progressive Conservative, 21.8% reported Liberal, 11% reported NDP, 13.2% opted for another party, while 29.6% of participants did not know who they would vote or refused to answer the question. In all, 57.6% of participants reported very good or excellent health, whereas 14.1% reported fair or poor health. More than 38% of participants reported having very good or excellent knowledge and understanding of the health issues affecting Ontarians.

**Table 1:** Characteristics of 2,006 Ontario adult participants surveyed in late 2010

		**% (N)**
**Age group**	18 to 34	28.2 (565)
	35 to 54	40.0 (803)
	55+	31.8 (639)
**Sex**	Male	48.0 (964)
	Female	52.0 (1042)
**Residence in a Census Metropolitan Area**^**1**^	Yes	72.7 (1458)
	No	27.3 (548)
**Place of birth and immigration status**	Born in Canada	76.0 (1493)
	Born outside Canada and immigrated >10 ago	19.4 (380)
	Born outside Canada and immigrated ≤10 ago	4.6 (91)
**Language spoken most often at home**	English	88.9 (1783)
	Non-English	10.3 (206)
**Visible minority**^**2**^	Yes	17.0 (321)
	No	83.0 (1568)
**Annual household income < $40,000**	Yes	29.8 (516)
	No	70.2 (1213)
**Educational attainment**		
**≤ high school diploma**	Yes	26.5 (524)
	No	73.5 (1455)
**Currently unemployed**	Yes	6.2 (122)
	No	93.8 (1856)
**If the election were being held today, would vote:**	Liberal	21.8 (437)
	New Democratic Party	11.0 (220)
	Progressive Conservative	24.5 (491)
	Other (incl. Green Party)	13.2 (264)
	Don’t know or refused	29.6 (594)
**Self-rated health**	Poor	3.7 (74)
	Fair	10.4 (209)
	Good	28.2 (565)
	Very good	36.8 (739)
	Excellent	20.8 (417)
**Knowledge and understanding of the health issues affecting Ontarians**	Poor	3.7 (75)
	Fair	16.6 (333)
	Good	40.7 (817)
	Very good	28.6 (573)
	Excellent	9.6 (193)

Almost 73% of Ontarians agreed with the general premise that all people are not equally healthy in Ontario (Table 2). Those participants with low educational attainment were more likely to be in disagreement than those with more than a high school diploma (RR 1.82, 95%CI 1.45–2.28), as were those who most often speak non-English languages at home compared to those who spoke English more regularly (RR 1.45, 95%CI 1.05–1.99). Fewer participants were aware of health inequalities between the rich and the poor in Ontario (Table 2). Almost 53% of participants agreed that the rich are much healthier than the poor, while 64.1% agreed that the poor are less likely to live into their eighties than the rich, and 56.5% agreed that rich people have been getting healthier relative to poor people recently. Older participants - especially those over 55 years of age - were consistently more aware of income inequalities than younger participants, while those living in urban areas, those politically affiliated with the NDP (compared to those who didn’t know who they would vote for or who refused to answer the question), and those who claimed to have at least a very good understanding of the health issues of Ontarians were often more likely to be aware of income inequalities.

**Table 2:** Percentage of participants reporting awareness of general health inequalities in Ontario, and relative risk of unawareness in subgroups

		**All people are not equally healthy**	**The rich are much healthier than the poor**	**The poor are less likely to live into their 80s**	**Rich people have been getting healthier relative to poor recently**
All participants (% in agreement with statement)		72.6	52.7	64.1	56.5
**Relative risk (95% confidence interval) of being unaware of health disparities**^**1**^					
Age (years):	18–34	-	Reference	Reference	Reference
	35–54	-	0.68 (0.54–0.86)	0.77 (0.61–0.97)	-
	55+	-	0.51 (0.40–0.65)	0.65 (0.50–0.83)	0.72 (0.56–0.93)
Male sex		-	0.78 (0.65–0.94)	-	-
Residence in a Census Metropolitan Area		-	0.71 (0.58–0.87)	0.75 (0.60–0.92)	-
Birth place and immigration status:	Born in Canada	-	-	-	Reference
	Immigrated 10+ years ago	-	-	-	0.73 (0.56–0.94)
	Recent immigrant	-	-	-	-
Non-English language spoken most often at home		1.45 (1.05–1.99)	-	-	1.64 (1.17–2.32)
Visible minority		-	-	-	-
Household income under $40,000		-	-	-	-
Low educational attainment		1.82 (1.45–2.28)	-	1.83 (1.47–2.28)	-
Currently unemployed	-	-	-	-	0.49 (0.32–0.74)
If the election were being held today, would vote:	Liberal	-	-	-	-
	New Democratic Party	-	0.56 (0.40–0.78)	-	0.67 (0.48–0.95)
	Progressive Conservative	-	-	-	-
	Other (incl. Green Party)	-	-	-	-
	Don’t know or refused	-	Reference	-	Reference
Fair or poor self-rated health		−0.62 (0.45–0.85)	-	-	-
Very good understanding of Ontarians’ health problems		0.75 (0.61–0.94)	0.82 (0.68–0.99)	0.74 (0.60–0.91)	-

Awareness of income-related inequalities in specific outcomes was considerably lower than awareness of health inequalities at-large, ranging from 18% for accidents to 35% for obesity (Table 3). The subpopulations that were more or less likely to be aware of such inequalities varied per condition; however, participants with low educational attainment were again often less likely to be aware of income-related inequalities compared to participants with higher educational attainment. On the other hand, males (compared to females) and those who indicated that they would vote for the Liberal Party in a current election (compared to those with no political affiliation) were often more likely to be aware of specific income-related inequalities. In fewer cases, older participants, participants living in urban areas, those not speaking English at home, or those with a very good understanding of the health issues affecting Ontarians were more likely to be aware of income-related inequalities.

**Table 3:** Percentage of participants reporting awareness of specific health inequalities by income in Ontario, and relative risk of unawareness in subgroups

		**The rich are less likely to suffer from…**								
		***Heart disease***	***Mental illness***	***Stress and anxiety***	***Depression***	***Alcoholism***	***Accidents***	***Diabetes***	***Obesity***	***Lung disease***
All participants (% in agreement with statement)		18.5	22.1	21.6	23.0	22.6	18.0	25.3	35.4	27.1
		**Relative risk (95% confidence interval) of being unaware of health disparities**^**1**^								
Age (years):	18–34	-	-	-	-	-	Reference	-	-	Reference
	35–54	-	-	-	-	-	0.64 (0.47–0.88)	-	-	0.56 (0.43–0.73)
	55+	-	-	-	-	-	0.53 (0.38–0.74)	-	-	0.43 (0.32–0.57)
Male sex		-	0.70 (0.57–0.87)	-	-	0.75 (0.61–0.93)	0.79 (0.62–1.00)	-	-	0.76 (0.62–0.94)
Residence in a Census Metropolitan Area		-	-	-		0.74 (0.58–0.96)	-	-	0.75 (0.61–0.93)	-	
Birth place and immigration status:	Born in Canada	-	-	-	-	-	-	-	-	-	
	Immigrated 10+ years ago	-	-	-	-	-	-	-	-	-	
	Recent immigrant	-	-	-	-	-	-	-	-	-	
Non-English language spoken most often at home		0.69 (0.49–0.98)	-	0.69 (0.49–0.97)	-	-	-	-	-	-	
Visible minority		-	-	-	-	-	0.67 (0.49–0.92)	-	-	-	
Household income under $40,000		-		0.77 (0.60–0.99)		-	-	-	-	-	
Low educational attainment		-	-	-		1.49 (1.15–1.93)	-	1.57 (1.22–2.03)	1.60 (1.28–2.01)	1.40 (1.10–1.79)	
Currently unemployed		-	-	-		-	-	-	-	-	
If the election were being held today, would vote:	Liberal	0.61 (0.44–0.84)	-	-	-	-	0.54 (0.38–0.77)	0.66 (0.50–0.88)	-	0.73 (0.55–0.97)	
	New Democratic Party	-	-	-	-	-	-	0.69 (0.48–0.98)	-	-	
	Progressive Conservative	-	-	-	-	-	0.66 (0.47–0.94)	-	-	-	
	Other (incl. Green Party)	-	-	-	-	-	0.64 (0.43–0.97)	-	-	-	
	Don’t know or refused	Reference	-	-	-	-	Reference	Reference	-	Reference	
Fair or poor self-rated health	-	-		0.67 (0.49–0.92)	-	-	-	-	-	-	
Very good understanding of Ontarians’ health problems	-	-	-	-	-	-	-	0.77 (0.62–0.95)	0.77 (0.63–0.93)	-	

## Discussion

In this study, we found that approximately 27% of this sample of Ontarians were unaware of income-related inequalities in health in the province. A similar proportion of the Canadian public (24%) was unable to identify particular groups of Canadians with worse health than others in a survey conducted in 2003 [31]. A smaller majority of participants in the current survey were *aware* of health inequalities by income level specifically (53%–64%). This is a much higher proportion than in the previous national study, where only 30% identified economically disadvantaged groups as having worse health than others [31]. More participants may have been aware of income-related health inequalities in our study compared to the national study because of how the question was framed (i.e., in the national study, this question appears to have been open-ended; whereas in our study, we directly asked participants about income-related health inequalities). Regardless of whether or not these results indicate that the level of awareness about income-related inequalities has grown over time, our study suggests that there remains a core minority of the population who are not aware of social inequalities in health generally. A rise in awareness about income-related health inequalities is plausible though, and could reflect the growing media coverage of social inequalities in relation to the recent “Great Recession” (officially from early 2008 to mid-2010). Conversely, a similar study conducted in the city of Saskatoon in 2004 (read: pre-Recession) found that 82% of residents agreed that “income affects how healthy we are” [28], which is a higher level of awareness than in our Ontario sample. This may reflect differences in the social environment of the city of Saskatoon and the province of Ontario. Increased awareness of health inequalities in Saskatoon may reflect a more urban study population (note that awareness was higher in participants living in CMAs in our study). It may also reflect historically popular familiarity with the concept of health inequalities. Tommy Douglas, the Premier of Saskatchewan from 1944 to 1961, led the first social democratic government in North America and is well-known for being the “father” of universal health care in Canada.

We also observed much lower awareness of income-related inequalities in specific health conditions. At worst, fewer than one in five Ontarians were aware of income-related inequalities in accidents and heart disease. These data suggest that even the small majority of Ontarians who indicated that the rich tend to be healthier than the poor in general have an incomplete understanding of the range of health outcomes in which there are income-related inequalities. Therefore, in addition to trying to reduce the size of the core minority who remain unaware of social inequalities in health, public awareness about the variety of income-related inequalities could also be improved. Again, awareness of condition-specific income-related inequalities was generally higher in the Saskatoon study across the four outcomes that were examined in both studies: mental illness, diabetes, heart disease and accidents. For example, 55% of Saskatoon residents were aware of inequalities in diabetes, compared to 25% of Ontarians in our study.

Our results suggest that there is a need for health equity advocates, physicians, researchers and public health authorities in Ontario to increase the effectiveness of knowledge translation activities for research studies that identify health inequalities, but also for major documents like the recent report of the Commission on Social Determinants of Health [32] that summarize the extent and nature of health inequalities. This echoes the call by the Federal, Provincial and Territorial Advisory Committee on Population Health in 1994 to strengthen population health and encourage the prioritization of action on health inequalities by strengthening public understanding about the broad determinants of health [33]. In this way, our study helps to inform a targeted strategy to improve awareness among specific subgroups of Ontarians. Such a strategy may include the use of social marketing strategies to ensure that evidence and analysis of health inequalities are translated with messaging that resonates with specific subpopulations who are less aware of these social differences in health [34-36]. For example, we consistently found lower awareness of income-related inequalities in easily identifiable demographic groups, like younger participants and those living in rural settings.

It is particularly interesting to note that participants in our study who had low educational attainment were often less aware than their more highly educated counterparts about health inequalities. This finding suggests that health inequalities rooted outside of the health care system are not inherently obvious to those who are negatively affected, and implies that fostering greater awareness among members of marginalized groups may help to empower these communities to better advocate for improvements in the health system.

Rigby et al. (2009) argue that awareness of health inequalities may be skewed by one's engagement with specific media sources that rarely discuss inequalities (Mutz, 1998) and by assumptions made about affected populations [24]. Morrison (2009) argues that the Canadian media ought to be engaged more with “local and regional public health actors” to increase awareness in the general population [37]. Unfortunately, several authors have noted a lack of interest in reporting on social determinants of health in the Canadian media [37,38], which represent one of the main conduits for evidence to be translated widely to the public. In particular, Raphael (2011) argues that the neglect of this topic in the mainstream media may be encouraged by “political and economic societal structures” [39], so the media have a responsibility to increase their own participation in facilitating dissemination of health inequalities to less aware populations. In particular, there may be opportunities for so-called “new media” technologies in the context of an increasingly media-saturated environment [34].

We also found recurrent differences in awareness by political affiliation. Participants who expressed an affiliation with the NDP or Liberal Party were often more likely to be aware of health inequalities than other participants. Given the social-democratic orientation of the NDP^d^, we hypothesize that participants who reported NDP affiliation may have a political ideology that acknowledges differences in quality of life across social classes, which could have led to greater awareness of social inequalities among these participants. Greater awareness of health inequalities among Liberal voters appears to reflect both a more highly educated population than other participants (18% of Liberals reported low educational attainment versus 29% of others; p < 0.0001), as well as greater knowledge of health issues affecting Ontarians (44% of Liberals reported a very good understanding versus 37% of others; p = 0.006). Moreover, Liberal voters of low educational attainment were comparatively more likely to report a very good understanding of health issues affecting Ontarians compared to other participants of low educational attainment (35% versus 24%, respectively; p = 0.05). These results indicate a need to increase awareness of social inequalities in health among Ontarians affiliated with other political parties, including the Progressive Conservative Party, as well as those who are “apolitical”.

More intensive research is required to understand why some sub-populations in our study had lower awareness of health inequalities, and these explanations may be complex [40]. In particular, there were contradictory findings related to socioeconomic position of participants where those of low education were often less likely to be aware of health inequalities compared to those with educational attainment beyond a high school diploma, while those with low income or who reported being unemployed were sometimes more likely to be aware relative to their higher socioeconomic position counterparts. Intriguingly, we also found consistent differences by sex, where females were often less aware of income inequalities than males. Our research team may also learn more about these differences as we continue to analyze attributions of the causes of health inequalities in the current survey.

On a related note, our continuing analysis of this Ontario survey with regard to both attribution as well as solutions for health inequalities will lead to a more complete understanding of how public opinion may influence political will to act on health inequalities in this setting. For example, if few Ontarians attribute income-related health inequalities to differential access to resources outside of the health care system (e.g., high quality, affordable housing), then increasing public awareness about such inequalities by itself may not lead to action on non-health care factors.

This study has several limitations. First, although telephone sampling is a powerful survey tool, inherent biases are present. Telephone interview surveys exclude households without conventional landlines, raising issues of representativeness. In Canada, there is evidence that those with lower socioeconomic position are more likely to opt for cellular telephones, thus restricting their likelihood of being sampled in our study [41]. Hence, our sample is relatively over-represented by individuals in the upper socioeconomic categories. In a similar way, our sample may be over-represented by participants from non-CMA (read: rural) parts of Ontario since cellular service may be more unreliable outside of CMAs. To combat any deviations from having as representative a sample as possible, our survey data was statistically weighted to be representative of Ontario adults in terms of composition by age and sex. Our study indicates that urban populations are more likely to be aware of health inequalities, but that those of lower educational attainment are likely to be less aware of some inequalities. In turn, we may expect a mixture of positive and negative bias due to these unintentional study exclusions.

Although the response rate in this study was low, it is typical of the response for RDD telephone interview surveys of this nature. A recent paper from the United States has noted the risks with plummeting response rates from RDD surveys, but the authors’ research into “nonresponse error in telephone surveys that focus on social and political topics” and recent meta-analyses suggest that “within the limits of the experimental conditions, nonresponse did not introduce substantial biases into the estimates” of the surveys explored [42]. That said, to combat this, a quota sample was constructed, meaning telephone numbers were randomly generated and sampled, and quotas for sex, age, and regional representation from the Ontario population were filled. The data were then further weighted by age and sex as per the 2006 Canadian Census.

Another limitation is related to our survey design. Differences in the level of awareness about health inequalities in general and income-related inequalities in Table 2 may partly reflect the different framing of these questions. Framing is known to have important effects on participant responses [43]. As suggested by the survey design literature [43], in order to minimize the likelihood that question framing would affect how participants interpreted issues, we repeated and/or modeled questions already found in similar surveys (e.g., [28]); we piloted the survey to qualitatively assess ease of comprehension; we attempted to ask straightforward questions, posing them in the positive wherever possible; and, we phrased questions concretely and in linguistically simple terms so as to make it easier for respondents to form a determined opinion.

## Conclusions

This is the first province-wide study in Canada, and the first in Ontario, to explore public opinion on social inequalities in health. Data from this first stage of analysis indicate that a majority of Ontarians recognize income-related inequalities in health, and suggest that there may have been an increase in public awareness since 2003. At the same time, almost one-third of this sample of Ontarians are unaware of income-related health inequalities and there is even less awareness of inequalities for certain health outcomes. This misalignment of public awareness about income-related inequalities in health with evidence produced by researchers in Ontario and Canada over the last decade can be seen as a barrier to the implementation of policies to support health equity in this setting and may contribute to the lack of political will to implement relevant policy change.

These findings indicate a need for public health advocates in Ontario to improve knowledge translation activities about health inequalities, including by targeting less aware populations and through greater participation of the media at-large. While the Ontario government has demonstrated a recognition of health inequalities, and while there are a range of feasible policy options for tackling structural and intermediate determinants of health [38,44,45], improving public awareness may be critical to foster the greater political will needed for action to address health inequality in Ontario.

## Endnotes

^a^ World Health Organization has defined health equity as circumstances under which no one is disadvantaged from achieving their full health potential due to socially determined factors [46]. ^b^ Although the Ontario government has formulated a long-term affordable housing strategy (Ministry of Municipal Affairs and Housing 2010) and recently passed the Strong Communities through Affordable Housing Act (Legislative Assembly of Ontario 2011), neither of these appear to include any mandate that wwill necessarily correct the under-supply of affordable housing, which will also require coordinated action from federal and municipal governments. ^c^ Although this is a large discrepancy, some of this differences is likely driven by a different minimum age included in our study population (as young as 18 years of age) and the comparison data from the 2006 Census [47], where data on educational attainment were reported among those at least 25 years of age. In this way, lower rates of high school completion in our study population may partly reflect younger individuals (i.e., below age 25) who have not yet completed high school, but who may still graduate before age 25. ^d^ The New Democratic Party was formed in 1961 after a merger of the Canadian Labour Congress and the Co-operative Commonwealth Federation.

## Competing interests

The author(s) declare that they have no competing interests.

## Authors’ contributions

KS designed and carried out the analysis and drafted the manuscript. AL and MK designed the analysis and critically revised the manuscript. CQ designed and supervised the analysis and critically revised the manuscript. All authors read and approved the final manuscript.
